# Diagnostic Accuracy of Imaging Modalities in the Evaluation of Vascular Invasion in Pancreatic Adenocarcinoma: A Meta-Analysis

**DOI:** 10.4021/wjon657w

**Published:** 2013-05-06

**Authors:** Angela E. Li, Bob T. Li, Bernard H.K. Ng, Sam McCormack, John Vedelago, Stephen Clarke, Nick Pavlakis, Jaswinder Samra

**Affiliations:** a Department of Radiology, Royal Prince Alfred Hospital, Camperdown NSW 2050, Australia; b Department of Medical Oncology, Royal North Shore Hospital, St Leonards NSW 2065, Australia; c Imaging Partners Online, Sydney NSW 2000, Australia; d Department of Gastrointestinal Surgery, Royal North Shore Hospital, St Leonards NSW 2065, Australia; e Sydney Medical School, University of Sydney, Camperdown NSW 2050, Australia

**Keywords:** Pancreatic neoplasms, Sensitivity and specificity, Tomography, X-ray computed, Endosonography, Meta-analysis

## Abstract

**Background:**

The extent of vascular invasion is a key factor determining the resectability of non-metastatic pancreatic adenocarcinoma. The purpose of this study is to determine the diagnostic accuracy of computed tomography (CT), endoscopic ultrasound (EUS), and magnetic resonance imaging (MRI) in the pre-operative evaluation of vascular invasion in pancreatic adenocarcinoma, with surgery as the reference standard.

**Methods:**

A search of the MEDLINE database for relevant articles in the English language published between January 2000 and February 2009 was performed. From each study, 2 × 2 tables were obtained, and pooled sensitivity, specificity, positive likelihood ratios, negative likelihood ratios and diagnostic odds ratios were calculated for each modality, along with a summary receiver operating characteristics (SROC) curve.

**Results:**

16 studies with a total of 797 patients who had surgical assessment of vascular invasion were included in the analysis. Several studies evaluated more than one imaging modality, allowing 24 datasets to be obtained in total. Sensitivity was highest for CT (0.73, 95% CI 0.67 - 0.79), followed by EUS (0.66, 95% CI 0.56 - 0.75) and MRI (0.63, 95% CI 0.48 - 0.77). The specificity for all three imaging modalities was comparable. The diagnostic odds ratios for CT, EUS and MRI were 45.9 (95% CI 18.0 - 117.4), 23.0 (95%CI 9.4 - 56.6), 23.9 (95% CI 5.4 - 105.1) respectively.

**Conclusion:**

CT was more accurate than EUS and MRI in the evaluation of vascular invasion in pancreatic adenocarcinoma and should be the first line investigation in pre-operative staging.

## Introduction

Pancreatic adenocarcinoma is the fourth leading cause of cancer death with mortality rates close to the incidence. At initial presentation, 80% of patients will have advanced disease, leaving only a minority patients suitable for resection [1]. Even with successful resection, the five-year survival is only 15-25% [2-5]. In the population of patients who undergo curative resection, the margin resection status, along with presence of nodal disease are significant predictors of survival [6, 7].

The role of pre-operative imaging is to select which patients are likely to have a margin-free resection, and therefore are likely to benefit from pancreaticoduodenectomy. Vascular invasion is a key factor in determining margin status and resectability [8]. Definitions of resectability are evolving with the advancement of surgical techniques, with greater importance placed on arterial rather than venous invasion [9]. The current definition of resectability includes absence of distant metastatic disease and absence of T4 tumour. A T4 tumour is defined as one which invades the celiac axis or superior mesenteric artery [10]. For head or body of pancreas tumors, the National Comprehensive Cancer Network guidelines define the following as being unresectable: distant metastases, greater than 180 degrees superior mesenteric artery encasement, any celiac abutment, unreconstructable superior mesenteric vein/portal vein occlusion, aortic invasion or encasement [11].

Methods used to assess the presence of vascular invasion include computed tomography (CT), endoscopic ultrasonography (EUS), and magnetic resonance imaging (MRI), laparoscopic ultrasound, and, intravascular ultrasonography. The optimal imaging modality to assess vascular invasion in pancreatic cancer has been debated. Some individual studies have shown EUS is superior to CT in predicting vascular invasion by pancreatic tumours [12, 13].Other authors recommend the use of CT as the first line investigation in staging of pancreatic cancer [14, 15].

There have been no previously published meta-analyses comparing the diagnostic performance of CT, EUS and MRI in assessment of vascular invasion in pancreatic cancer. The aim of this study was to perform a meta-analysis comparing the diagnostic accuracy of modern CT, EUS, and MRI in predicting vascular invasion in patients who undergo surgery for pancreatic adenocarcinoma.

## Methods

### Search strategy

A MEDLINE literature search was conducted to identify articles published in the English language from January 2000 to February 2009, pertaining to EUS, CT, or MRI evaluation of vascular invasion in pancreatic cancer. Search terms that were used included combinations of ‘pancreatic neoplasm’ (MeSH), ‘pancreatic adenocarcinoma’, ‘pancreatic cancer’, ‘endoscopic ultrasonography’, ‘endosonography’, ‘EUS’, ‘endoscopic ultrasound’, ‘Tomography, X-Ray Computed’ (MeSH), ‘computed tomography’, ‘CT’, ‘Magnetic Resonance Imaging’ (MeSH), ‘magnetic resonance’, ‘MRI’, or ‘MR imaging’, ‘neoplasm staging’ (MeSH), ‘vascular’, ‘vessel’, ‘arterial’, ‘artery’, ‘venous’, and ‘vein’. These were combined with a search for articles relating to diagnostic accuracy using the search terms ‘Sensitivity and Specificity’ (MeSH), ‘Predictive Value of Tests’ (MeSH), ‘predictive value’, ‘sensitivity’ and ‘specificity’. Additional studies were also obtained from the reference lists of primary studies and review articles.

### Study eligibility

Studies were included if they met the following criteria: published in the English language, the study population consisted of patients being investigated for suspected pancreatic adenocarcinoma who underwent pre-operative evaluation of vascular invasion with EUS, CT or MRI. Furthermore, only the subgroup of patients who had surgery to confirm or refute the presence of vascular invasion was included in the meta-analysis. This group consisted of patients who underwent curative resection, palliative or explorative surgery.

Studies were excluded if the results of the vascular invasion were not reported separately (from other criteria for resectability), there was insufficient information on the definition of vascular invasion used, a 2 × 2 table could not be obtained, there was a potentially overlapping study population, or if the results were reported for individual vessels and could not be obtained on an individual patient basis.

Studies were also excluded if they did not meet the following criteria for the definition of vascular invasion. For EUS, criteria for vascular invasion included of loss of the hyperechoic interface between tumour and vessel, tumour in the vessel lumen, or collaterals associated with venous occlusion [16, 17]. The CT and MRI criteria for vascular invasion included irregularity of the vessel wall, vascular compression or apposition with concavity toward vessel lumen, vascular encasement greater than 180°, vascular thrombosis or tumour in the lumen, or presence of collateral vessels.

### Data extraction

Data was extracted independently by two readers using a standardised proforma, and any discrepancies were resolved by consensus. The readers were not blinded to the names of the authors or journal of publication. The following data was collected: year of publication, sample size and number of patients who had surgical staging, mean age, sex, imaging modality evaluated, characteristics of study quality, and study results. The number of true positives, false positives, true negatives and false negatives were extracted to form a 2 × 2 table for each study.

### Statistical analysis

Data was analysed separately for CT, EUS, and MRI. From the 2 × 2 contingency tables, sensitivity and specificity were determined for individual studies. Pooled sensitivity, specificity, positive likelihood ratio and negative likelihood ratio, along with the respective 95% confidence intervals (CI) were calculated. A value of 0.5 was added to all cells of studies that contained a count of zero to avoid potential problems in odds ratio calculations for studies with sensitivities or specificities of 100%. The diagnostic odds ratio (DOR) which is a summary of diagnostic performance was also calculated. The DOR is the ratio of odds of a positive test in patients with the disease to the odds of a positive test in patients without the disease [18].

Heterogeneity was assessed with the Cochran’s Q test using a random effects model (DerSimonian and Laird) and the I-square (I^2^) statistic. A P-value of less than 0.05 by Cochran’s Q test indicates significant heterogeneity. The I^2^ statistic indicates the percentage of variation in study results which are due to heterogeneity rather than chance. An I^2^ statistic of 0% indicates no observed heterogeneity, with larger percentages corresponding to greater heterogeneity [19]. As improvements in CT technology have led to improved resolution which may affect the accuracy when evaluating vascular invasion, a subgroup analysis comparing single slice CT with multiple detector CT (MDCT) was performed.

Summary receiver operating characteristic (SROC) analysis was conducted to account for the interdependence between sensitivity and specificity, using the Moses and Littenberg model and a weighted area under the curve (AUC) [20]. From the SROC curve, the Q* point was determined. Q* is the point on the SROC curve where sensitivity equals specificity. This indicates how accurate a test is compared to ideal test where sensitivity and specificity are both 100%.

Meta-regression was conducted to examine the effect of year of publication, sample size, mean patient age, gender distribution, and study design on the estimates of diagnostic accuracy. The threshold effect was tested using the regression equation D = *a* + *b*S, with the absence of a threshold effect indicated by b = 0 and P > 0.05. Statistical analyses were performed using Metadisc software. (Version 1.4; Clinical Biostatistics Unit, Ramon y Cajal Hospital, Madrid, Spain).

## Results

### Literature search results

The MEDLINE search yielded 212 studies, and an additional 9 studies were identified through searching of reference lists. One hundred and thirty-six studies were excluded on the basis of title or abstract. Eighty-five potentially eligible articles were assessed according to the full selection criteria. Studies were excluded as they did not specifically assess vascular invasion (11), had an inadequate reference standard (5), did not meet the criteria for the definition of vascular invasion (8), contained a potentially overlapping study population (1), or if the vascular invasion results were not reported separately from results of overall resectability (22). Studies were also excluded if a 2 × 2 table could not be obtained (10), or results could not be obtained on an individual patient basis (12).

Sixteen studies satisfied the selection criteria, and the study characteristics are outlined in Table 1 [12, 13, 21-34]. The 16 studies included a total of 1,070 patients, of which 797 patients had surgical assessment of vascular invasion. The numbers of studies which evaluated CT, EUS, and MRI were 12, 8 and 4 respectively, with 6 studies evaluating multiple imaging modalities. The study by Mertz et al evaluated EUS and CT, but the definition of vascular invasion was only given for EUS. As there was insufficient information on the definition of vascular invasion for CT, only the results of EUS were included in this meta-analysis.

**Table 1:** Characteristics of Included Studies

	Year	Study design	Patients (n)	Mean age (years)	Males (%)	Modality
Mertz [12]	2000	P	16	NS	NS	EUS
Ahmad [21]	2001	P	21	61	76	EUS
Arslan [22]	2001	R	31	63	48	CT, MRI
Tierney [13]	2001	P	24	64	57	CT, EUS
Lopez Hanninen [23]	2002	P	34	58	55	MRI
Procacci [24]	2002	R	63	64	59	CT
Valls [25]	2002	P	39	61	62	CT
Rivadeneira [26]	2003	R	44	62	58	CT, EUS
Yusoff [27]	2003	R	32	60	62	EUS
Ramsay [28]	2004	P	19	57	44	CT, EUS, MRI
Soriano [29]	2004	P	59	65	53	CT, EUS, MRI
Vargas [30]	2004	R	25	64	52	CT
Karmazanovsky [31]	2005	R	69	60	58	CT
Li [32]	2005	P	54	61	67	CT
Buchs [33]	2007	R	153	NS	49	CT, EUS
Zamboni [34]	2007	R	114	70	46	CT

n: number of patients who had surgical staging; NS: not specified; P: prospective; R: retrospective.

The mean patient age in the studies ranged from 57 to 70. The proportion of males in the studies ranged between 44-76%.

### Diagnostic performance

**Figure 1:**
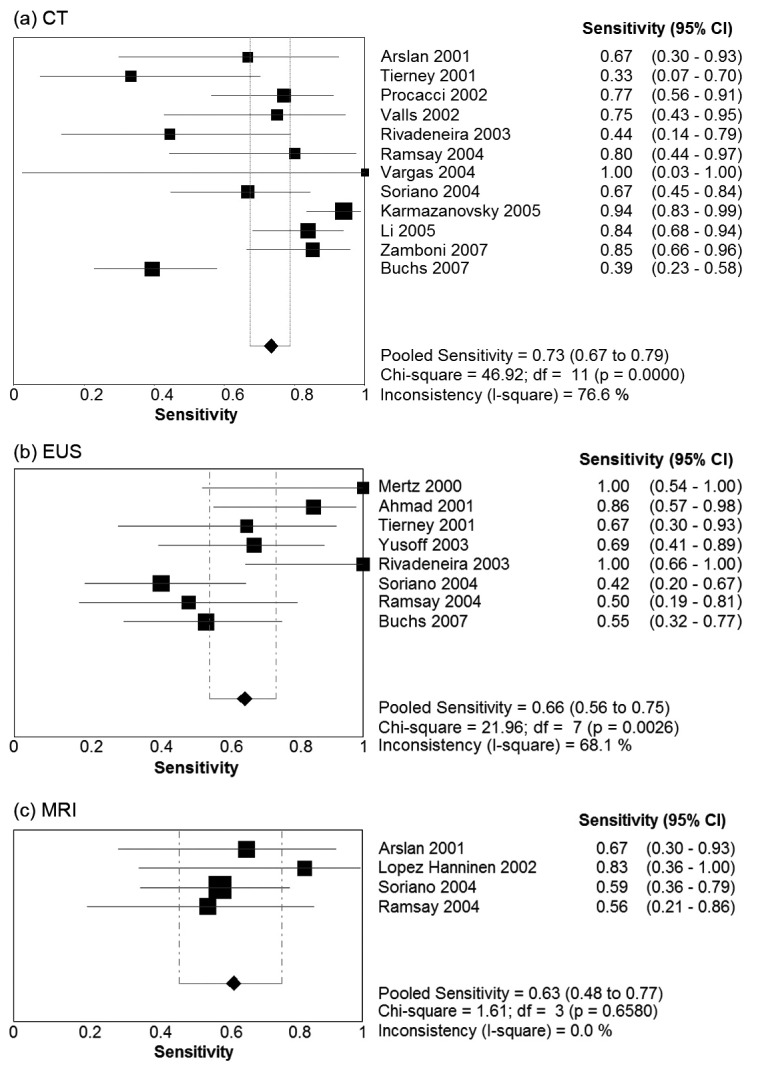
Forest plots of sensitivity Pooled results for sensitivity of (a) CT, (b) EUS, and (c) MRI in detection of vascular invasion in pancreatic adenocarcinoma. The limits of the diamond represent the 95% confidence interval of the pooled estimate.

**Figure 2:**
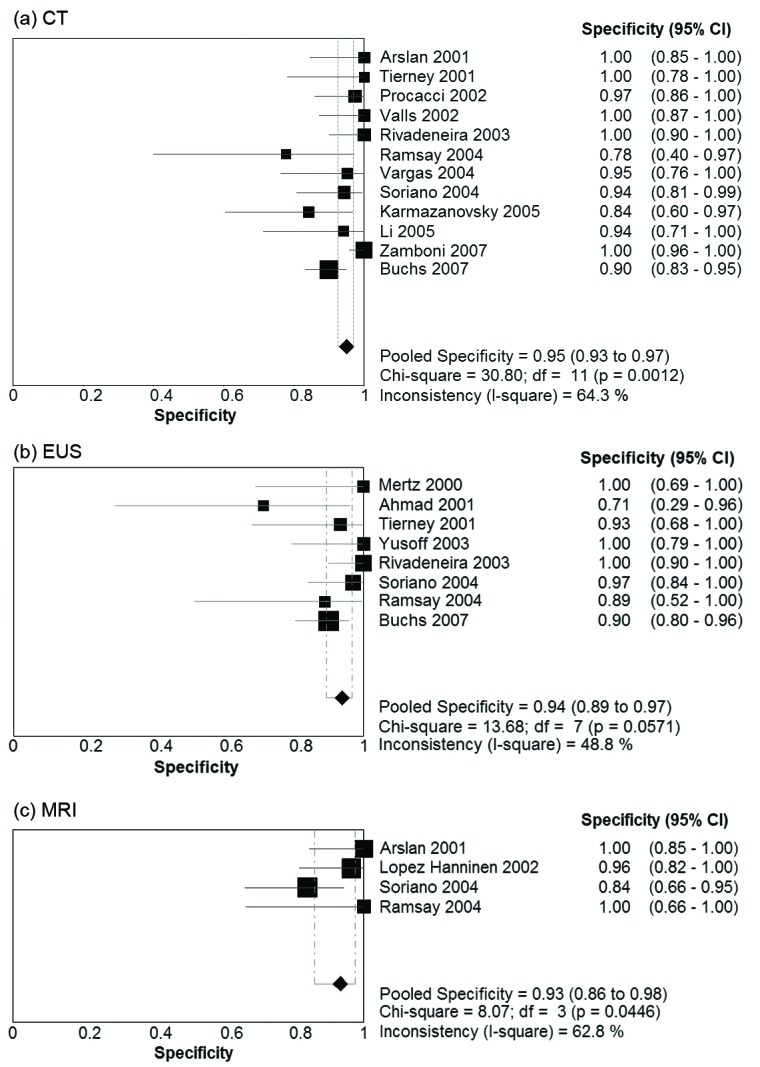
Forest Plots of specificity Pooled results for specificity of (a) CT, (b) EUS, and (c) MRI in detection of vascular invasion in pancreatic adenocarcinoma. The limits of the diamond represent the 95% confidence interval of the pooled estimate.

Table 2 presents summary estimates for the diagnostic performance of CT, EUS, and MRI in the evaluation of vascular invasion in pancreatic carcinoma. The pooled sensitivity was highest for CT, with a sensitivity of 0.73 (95% CI 0.67 - 0.79). EUS and MRI had sensitivities of 0.66 (95% CI 0.56 - 0.75) and 0.63 (95% CI 0.48 - 0.77) respectively. Specificity was comparable for the three imaging modalities: 0.95 (95% CI 0.93 - 0.97) for CT, 0.94 (95% CI 0.85 - 0.97) for EUS, and 0.93 (0.86 - 0.98) for MRI. Forest plots for sensitivity and specificity of the three imaging modalities are presented in Figure 1 and Figure 2. There was no evidence of a threshold effect for studies that evaluated CT (b = -0.04, P = 0.89), EUS (b = -0.043, P = 0.90), or MRI (b = -1.08, P = 0.29).

**Table 2:** Estimates of Diagnostic Accuracy

	No. Studies	Sensitivity (95% CI)	Specificity (95% CI)	LR+ (95% CI)	LR- (95% CI)	DOR (95% CI)	AUC (SE)	Q*
CT	12	0.73 (0.67 - 0.79)	0.95 (0.93 - 0.97)	10.41 (5.50 - 19.70)	0.31 (0.21 - 0.48)	45.94 (17.98 - 117.40)	0.93 (0.04)	0.87
EUS	8	0.66 (0.56 - 0.75)	0.94 (0.85 - 0.97)	7.02 (3.78 - 13.06)	0.42 (0.29 - 0.61)	23.03 (9.37 - 56.61)	0.89 (0.05)	0.82
MRI	4	0.63 (0.48 - 0.77)	0.93 (0.86 - 0.98)	8.88 (2.74 - 28.79)	0.44 (0.30 - 0.63)	23.89 (5.43 - 105.09)	0.65 (0.21)	0.62

CI: confidence interval; LR+: positive likelihood ratio; LR-: negative likelihood ratio; DOR: diagnostic odds ratio; AUC: area under the curve; SE: standard error; Q*: Cochran’s Q point; the point on the SROC curve where sensitivity equals specificity.

Estimates of diagnostic accuracy which take into account the interdependence between sensitivity and specificity demonstrated CT had the strongest diagnostic performance. The diagnostic odds ratio (DOR) was 45.9 for CT, 23.0 for EUS, and 23.9 for MRI. The SROC area under the curve (AUC) for CT, EUS and MRI was 0.94, 0.89, and 0.65 respectively. The Q* point, representing the highest joint sensitivity and specificity on the SROC curve was highest for CT (Q* = 0.87), followed by EUS (Q* = 0.82) and then MRI (Q* = 0.62).

### Heterogeneity

There was significant heterogeneity in the DOR for studies pertaining to CT (Cochran’s c^2^ = 23.7, P = 0.01, 11 df, I^2^ = 53.6%). No significant heterogeneity in the DOR was observed for EUS (c^2^ = 8.4, P = 0.3, 7 df, I^2^ = 17.1%) or MRI (c^2^ = 4.5, P = 0.2, 3 df, I^2^ = 33.9%). The possible sources of heterogeneity for CT include the use of slightly different definitions for vascular invasion, and differences in CT scanner characteristics with some studies utilising single slice CT and other studies using MDCT. Furthermore, tests for heterogeneity have greater power to detect heterogeneity as the number of studies increases. Power to detect heterogeneity in the EUS and MRI subgroups was decreased due to the smaller number of studies.

### Subgroup analysis

The subgroup analysis included 9 studies that utilised single-slice CT, compared to the 4 studies that utilised MDCT. MDCT had a higher pooled sensitivity of 0.80 (95% CI 0.70 - 0.89) and specificity of 0.97 (95% CI 0.93 - 1.00). The diagnostic odds ratio for MDCT and single slice CT was 65.1 (95% CI 9.9 - 428.9) and 32.2 (11.5 - 90.2) respectively. Results of this subgroup analysis are given in Table 3.

**Table 3:** Subgroup Analysis: Single Slice CT Compared to MDCT

	No. Studies	Sensitivity (95% CI*)	Specificity (95% CI)	DOR (95% CI)
Single slice CT	9	0.70 (0.63 - 0.77)	0.94 (0.91 - 0.97)	32.21 (11.5 - 90.2)
MDCT	4	0.80 (0.70 - 0.89)	0.97 (0.93 - 1.00)	65.1 (9.89 - 428.88)

CI: confidence interval; DOR: diagnostic odds ratio.

### Meta regression

To explore sources of heterogeneity, meta-regression was conducted for the 24 datasets. The mean age, gender distribution, year of publication, method of data collection, and year of publication did not have a significant effect on the diagnostic odds ratio (P > 0.05).

## Discussion

This meta-analysis demonstrates CT has the highest diagnostic accuracy for assessment of vascular invasion in pancreatic adenocarcinoma, followed by EUS and MRI.

Our results differ from a meta-analysis by Puli et al, which contained 29 studies (N = 1,308) published between 1988 - 2005, evaluating endoscopic ultrasonography in the diagnosis of vascular invasion in pancreatic cancer [35]. This meta-analysis yielded a pooled sensitivity of 73%, specificity of 92%, and diagnostic odds ratio of 40.1 for EUS evaluation of vascular invasion. They conducted a subgroup analysis to assess the accuracy of EUS within different time periods and found that the newer studies demonstrated decreased diagnostic accuracy. The studies published between the year 2000 and 2005 had a pooled sensitivity of 66%, specificity of 86% and diagnostic odds ratio of 17.7. The authors suggested a possible reason for the reported higher diagnostic accuracy during earlier periods is the small number of studies performed in earlier periods compared to studies performed in later years [35]. This study was limited to EUS so comparisons to other imaging modalities cannot be made.

Bipat et al compared 68 studies using ultrasonography, CT and MRI in evaluating the resectability of pancreatic adenocarcinoma [36]. The pooled results for diagnostic accuracy encompassed a broad definition of unresectability which included any of the following: presence of portal venous invasion, lymph nodes metastases, or liver metastases. Results pertaining to vascular invasion were not reported separately. Dewitt et al reviewed 9 studies using both CT and EUS in the detection, staging and resectability of pancreatic cancer [37]. This included vascular invasion, although the definitions used for vascular invasion were not given. These authors did not pool results of diagnostic accuracy because they felt the studies were heterogeneous.

CT technology has evolved dramatically, initially with development of helical CT, followed by MDCT which has markedly improved resolution and decreased scanning time. Post-processing techniques such as multiplanar reconstructions (MPR), curved planar reformations, volume rendering and maximum intensity projections allow visualization of vessels in multiple formats [38]. Curved planar reformations generate longitudinal cross sections along vessels to assist in evaluating vessel invasion. Vargas et al demonstrated the use of curved planar reformations for MDCT led to correct assessment of vessel invasion in 109/110 vessels, and overall negative predictive value of 87% on a per vessel basis [30].

Our study showed MDCT had higher diagnostic accuracy than single-slice CT, although strong conclusions cannot be made due to the wide confidence intervals obtained. The subgroup analysis was limited by the small number of studies in the MDCT group (n = 4). Furthermore, most of the MDCT studies in this review used 4 and 8 slice CT scanners whereas higher resolution 64-slice scanners are more commonly used for pancreatic cancer staging today. Additional improvements in CT technology with wider area detectors allow larger volume imaging and shorter image acquisition time, decreasing variation in contrast enhancement and optimising image quality [39]. Use of dual source CT can also improve conspicuity of pancreatic adenocarcinomas from the normal pancreas in the portal venous phase [40].

Heterogeneity among individual studies was observed for the estimates of diagnostic accuracy. Reasons for heterogeneity were explored using meta-regression. However, study size or design, patient characteristics, of year or publication did not have a significant effect on the diagnostic odds ratio. Differences in imaging equipment and scanning protocols may have contributed to heterogeneity of results. Another possible contributor to heterogeneity is the use of different definitions for vascular invasion. We tried to overcome the effect of different thresholds resulting in varying estimates of diagnostic accuracy by using methods such as SROC analysis and diagnostic odds ratios. Furthermore, threshold analysis did not demonstrate a significant threshold effect.

Studies published prior to the year 2000 were excluded from this meta-analysis. This resulted in fewer studies being available for analysis. We felt limiting the time period was necessary to allow assessment of modern imaging technology.

### Conclusion

The results of this meta-analysis demonstrate that CT has a higher diagnostic accuracy than EUS and MRI in determining presence of vascular invasion in pancreatic adenocarcinoma. Based on these results, we recommend CT as a first line investigation in the pre-operative staging of patients with suspected pancreatic adenocarcinoma. If the CT is equivocal, EUS or MRI may be performed. As CT technology is rapidly advancing with increases detector number and width, improved contrast bolus timing and enhancement, and the use of dual energy levels, future studies may reveal additional improvements in the diagnostic accuracy of CT in evaluating arterial or venous invasion.
